# Energy transfer through third‐grade fluid flow across an inclined stretching sheet subject to thermal radiation and Lorentz force

**DOI:** 10.1038/s41598-023-46428-x

**Published:** 2023-11-10

**Authors:** Najiba Hasan Hamad, Muhammad Bilal, Aatif Ali, Sayed M. Eldin, Mohamed Sharaf, Mati Ur Rahman

**Affiliations:** 1 Building and Construction Department, Shaqlawa Technical College, Erbil Polytechnic University, Erbīl, Iraq; 2 Sheikh Taimur Academic Block-II, Department of Mathematics, University of Peshawar, Peshawar, 25120 Khyber Pakhtunkhwa Pakistan; 3 School of Mathematical Sciences, Jiangsu University, Zhenjiang, 212013 Jiangsu China; 4 Center of Research, Faculty of Engineering, Future University in Egypt, New Cairo, 11835 Egypt; 5 Industrial Engineering Department, College of Engineering, King Saud University, P.O. Box 800, 11421 Riyadh, Saudi Arabia; 6 Department of Computer Science and Mathematics, Lebanese American University, Beirut, Lebanon

**Keywords:** Energy science and technology, Engineering, Mathematics and computing, Physics

## Abstract

The heat and mass transfer through the third grade fluid (TGF) flow over an inclined elongating sheet with the consequences of magnetic field and chemical reaction is reported. The impact of activation energy, heat source/sink, and thermal radiation is considered on the TGF flow. Fluid that demonstrate non-Newtonian (NN) properties such as shear thickening, shear thinning, and normal stresses despite the fact that the boundary is inflexible is known as TGF. It also has viscous elastic fluid properties. In the proposed model, the TGF model is designed in form of nonlinear coupled partial differential equations (PDEs). Before employing the numerical package bvp4c, the system of coupled equations are reduced into non-dimensional form. The finite-difference code bvp4c, in particular, executes the Lobatto three-stage IIIa formula. The impacts of flow constraints on velocity field, energy profile, Nusselt number and skin friction are displayed through Tables and Figures. For validity of the results, the numerical comparison with the published study is performed through Table. From graphical results, it can be perceived that the fluid velocity enriches with the variation of TGF factor and Richardson number. The heat source parameter operational as a heating mediator for the flow system, its influence enhances the fluid temperature.

## Introduction

The fluid flow through a stretching sheet holds substantial significance within the domain of fluid dynamics, owing to its wide array of uses in various engineering and industrial domains. Abolbashari et al.^1^ analytically examined the behavior of fluid flow. Their study focused on a flow scenario where a stretching sheet was involved with velocity slip condition. Zeeshan et al.^2^ deliberated the heat transmission on the motion of a ferromagnetic fluid over an extending surface. This ferromagnetic fluid consisted of a well-blended mixture of magnetic solid particles all of this occurs in the presence of an electromagnetic dipole state. Shit et al.^3^ conducted a study that explored the dynamics of unsteady boundary layer magnetohydrodynamic flow and convective heat source. Sandeep and Sulochana^4^ developed a new mathematical model to investigate energy and heat transmission in non-Newtonian fluids on a stretched surface. Results showed that the Jeffrey nanofluid outperformed Maxwell and Oldroyd**-**B nanofluids in terms of heat transfer. Besthapu et al.^5^ conducted an examination of velocity slip on a extending sheet with convectively non-uniform characteristics. Alqahtani et al.^6^ conducted a 3D simulation on MHD behavior of hybrid fluid flow across double stretching surfaces. Chu et al.^7^ investigated a 2D continuous laminar movement of a TGF past a flow over a shrinking surface containing gyrotactic microorganisms. The flow was electrically conductive due to an applied electric field and the Buongiorno nanoliquid model was used for mathematical modeling. The study also incorporated chemical reactions with activation energy effects. Kumar et al.^8^ and Li et al.^9^ investigated the energy transmission rate in a hydromagnetic Williamson nanoliquid flow thru a absorbent strained sheet. Khan et al.^10^ conducted a discussion on the hybrid nanoliquid flow consisting of Cu and ${\text{Al}}_{2} {\text{O}}_{3}$ nanoparticles in water. This flow occurred from a centrifugally porous surface that could either shrink or stretch. Elattar et al.^11^ scrutinized the steady flow of hybrid nanoliquid over an impermeable stretchable sheet. A mathematical model was developed with the aim of improving the rates of energy transference, enhancing the efficiency and effectiveness of thermal energy propagation. Dogonchi et al.^12^ described the entropy and thermal analyses of the nanoliquid flow within a porous cylinder. Some remarkable results recently presented by Ref.^13–18^.

The flow of a mixture of fluid and solid particles is inherently complex and can be influenced by numerous variables. To better understand and study these intricate flows, one common approach is to treat the mixture as a NN fluid. Considerable research has been dedicated to the analysis of various transport phenomena occurring within non-Newtonian fluids, including substances like coal slurries. Among these processes, heat transfer is of particular significance in the context of handling and processing these fluids. It plays a pivotal function in the efficient management and treatment of such complex mixtures^19^. Ariel^20^ conducted a study on the laminar flow and steady of a TGF over a permeable flat conduit. Ellahi and Riaz^21^ carried out an investigation to examine the TGF with changing viscosity in a conduit. This study also considered the heat diffusion features of the fluid in the context of the analysis. Bilal et al.^22^ explored the MHD motion of Carreau Yasuda liquid initiated by an exponentially extending surface. Adesanya et al.^23^ performed a study on the intrinsic irreversibility linked with the motion of third-grade fluid through a conduit exposed to convective heating. This study recognizes that the heat generated leads to continuous entropy generation within the channel. Reddy et al.^24^ conducted an investigation to understand the effect of the Prandtl number on TGF around a vertically oriented cylinder that is uniformly heated. Mahanthesh and Joseph^25^ examined the steady-state behavior of third-grade liquid flowing over a pressure-type die in the existence of nanoparticles. The fluid is dissipative and its properties are considered to be constant throughout the analysis. Contemporary and innovative literature concerning Non-Newtonian (third-grade fluid) can be found in Refs.^26–30^.

Magnetohydrodynamics (MHD) is the discipline that analyzes the behavior of electrically conductive substances including plasmas, ionized gases and liquid metals when subjected to magnetic fields. This area of research investigates the interaction between fluid motion and electromagnetic forces and it possesses extensive applications in geophysics, engineering, plasma physics and astrophysics. The impact of fluctuating viscous flow within a narrowing channel was scrutinized by Al-Habahbeh et al.^31^. Rashidi et al.^32^ provided an extensive overview of the utilization of MHD and biological systems. The investigation of MHD fluid motion in diverse orientations linked to human anatomical structures is a significant scientific domain given its relevance and applications in the field of medical sciences. Ellahi et al.^33^ examined the concurrent impacts of MHD, heat transfer and slip over a flat plate in motion. Furthermore, this study also assessed the influence of entropy generation within this context. Lv et al.^34^ explored the effects of various physical phenomena, including diffusion-thermo, radiation-absorption in the context of MHD free convective spinning flow of nanoliquids. Kumam et al.^35^ studied the MHD Radiative unsteady fluid flow with the upshot of heat source across a channel placed in absorbent medium. Tian et al.^36^ studied the energy transfer though fluid flow surrounded by a rectangular enclosure having a heat sink filled with hybrid nanofluids and the exploration focused on the joint effects of forced and natural convection. Bhatti et al.^37^ conducted research into the unsteady flow within the confines of parallel spinning spherical disks placed in a permeable medium. The influence of magnetization on lubrication had attracted consideration due to their important roles in various industrial applications. One notable example was their increased use in high-temperature bearings with liquid metal lubricants. Alharbi et al.^38^ carried out a computational examination of the influence of different geometric factors on an extending cylinder. Hamid and Khan^39^ investigated the upshot of magnetic flux on NN Williamson fluid flow. The flow was induced by an elongating cylinder in the existence of nanocomposites. Shamshuddin et al.^40^ studied the upshot of chemical reactions Couette-Poiseuille nanoliquid flow through a gyrating disc. Kumam et al.^41^ explored the MHD unsteady radiative flow. Khan and Alzahrani^42^ focused on optimizing entropy and understanding heat transport in the flow of a magneto-nanomaterial. This investigation took into account the influence of MHD within the fluid. Adnan and Ashraf^43^ and Li et al.^44^ evaluated the nanoliquid flow across a permeable surface.

The originality of the proposed model is to examine the heat and mass transfer through the TGF flow over an inclined elongating sheet. The impact of magnetic field, activation energy and thermal radiation is considered on the TGF flow. Fluid that demonstrate NN properties such as shear thickening, shear thinning, and normal stresses despite the fact that the boundary is inflexible is known as TGF. In the proposed model, the TGF model is conveyed in form of nonlinear coupled PDEs. Before employing the numerical package bvp4c, the system of coupled equations are reduced into non-dimensional form. The significances of flow factors on velocity field, energy profile and Nusselt number are presented through Tables and Figures. For validity of the results, the numerical comparison with the published existing study is performed through Table. In the upcoming section, the problem is designed in form of PDEs and numerically solved.

## Formulation of the problem

**Figure 1:**
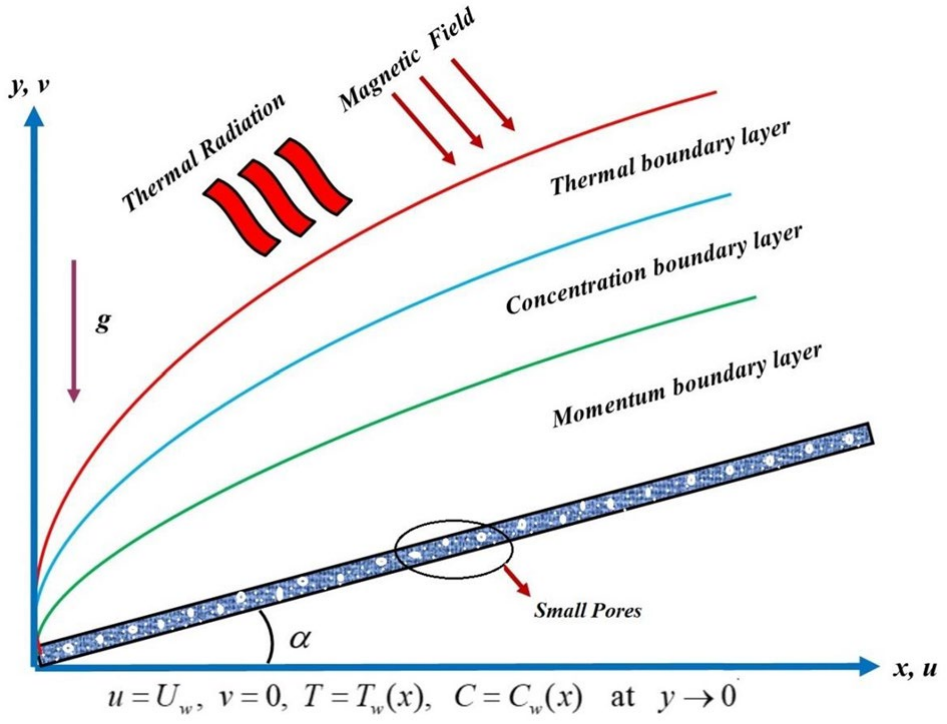
TGF flow across a stretching inclined surface.

We have considered the mass and energy transfer through the steady and incompressible flow TGF over an inclined elongating sheet. The two-dimensional TGF flow is inspected under the impacts of chemical reaction, magnetic field, activation energy and thermal radiation. The surface of the sheet is assumed to be Darcy permeable. The *x*-axis and *y-*axis is the horizontal and normal axis to an inclined stretching sheet as shown in Fig. 1. Here, *g, T*_*w*_ and *C*_*w*_ is the gravitational acceleration, surface temperature and concentration respectively. By keeping in view, the above suppositions, the TGF flow equations are expressed as^45, 46^:

1 $$\frac{\partial u}{{\partial x}} + \frac{\partial u}{{\partial x}} = 0,$$

2 $$\left. \begin{array}{ll} u\frac{\partial u}{{\partial x}} + v\frac{\partial u}{{\partial y}} = \nu \frac{{\partial^{2} u}}{{\partial y^{2} }} + \frac{{\alpha_{1} }}{\rho }\left( {u\frac{{\partial^{3} u}}{\partial y\partial x} + \frac{\partial u}{{\partial x}}\left( {\frac{{\partial^{2} u}}{{\partial y^{2} }}} \right) + 3\frac{\partial u}{{\partial y}}\left( {\frac{{\partial^{2} u}}{{\partial y^{2} }}} \right) + \nu \frac{{\partial^{3} u}}{{\partial y^{3} }}} \right) + 2\frac{{\alpha_{1} }}{\rho }\frac{\partial u}{{\partial y}}\left( {\frac{{\partial^{2} u}}{{\partial y^{2} }}} \right) + 6\beta_{3} \hfill \\ \left( {\frac{\partial u}{{\partial y}}} \right)\left( {\frac{{\partial^{2} u^{2} }}{{\partial y^{2} }}} \right) + g\left( {T - T_{\infty } } \right)\beta_{T} \cos \alpha + g\left( {C - C_{\infty } } \right)\beta_{c} \cos \alpha - \frac{{\alpha B(x)^{2} }}{\rho } - \frac{u\nu }{{K_{0} }} - Fu^{2} , \hfill \\ \end{array} \right\}$$

3 $$u\frac{\partial T}{{\partial x}} + v\frac{\partial T}{{\partial y}} = \alpha \frac{{\partial^{2} T}}{{\partial y^{2} }} + \frac{{Q_{0} }}{{\rho C_{p} }}\left( {T - T_{\infty } } \right) - \frac{1}{{\rho C_{p} }}\frac{\partial qr}{{\partial y}},$$

4 $$u\frac{\partial C}{{\partial x}} + v\frac{\partial C}{{\partial y}} = D_{m} \frac{{\partial^{2} C}}{{\partial y^{2} }} - k_{r}^{2} \left( {C - C_{0} } \right)\left( {\frac{T}{{T_{\infty } }}} \right)^{n} \exp \left( { - \frac{{E_{a} }}{\kappa T}} \right).$$

Boundary conditions (BCs) are^45^:

5 $$\left. \begin{array}{ll} u = U_{w} = U_{0} e^{\frac{x}{L}} ,\,\,\,C = C_{w} (x) = C_{\infty } + C_{0} e^{\frac{x}{L}} ,\,\,\,T = T_{w} (x) = T_{\infty } + C_{0} e^{\frac{x}{L}} ,\,\,\,v = 0\,\,\,\,\,{\text{at}}\,\,\,\,y \to 0 \hfill \\ u \to 0,\,\,\,\,C \to C_{\infty } ,\,\,\,T \to T_{\infty } \,\,\,\,{\text{at}}\,\,\,\,y \to \infty . \hfill \\ \end{array} \right\}$$

Here, ($\alpha_{1}$,$\beta_{3}$,$\alpha_{2}$) are the moduli of material. $U_{w}$ and $\delta$ is the sheet stretching velocity and electrical conductivity, $qr = - \frac{{4\sigma^{*} }}{{3k^{*} }}\frac{{\partial T^{4} }}{\partial y}$,$K_{0}$ and $Q_{0}$ is the thermal radiation, surface penetrability and heat source. $B(x) = B_{0} e^{\frac{x}{L}}$ and *Ea* is the magnetic field and activation energy, $k_{r}$ and $C_{p}$ is the chemical reaction and specific heat competence, $F = \frac{{C_{b} }}{{\sqrt {K_{0} } }}$,$\nu$ and $\mu$ is the inertia constant, kinematic and dynamic viscosity, $\alpha_{m}$ and $D_{m}$ is the mass and thermal diffusivity.

In order to simplify Eqs. (2)–(4) and (5) to nonlinear ODEs, we use the following makeover as^39^:

6 $$u = U_{0} e^{\frac{x}{L}} ,v = \sqrt {\frac{{\nu U_{0} }}{2L}} \left( {f(\eta )) + \eta f^{\prime}(\eta )} \right)e^{\frac{x}{L}} ,\,\,\,\eta = \sqrt {\frac{{U_{0} }}{2L\nu }} ye^{\frac{x}{L}} ,\,\,\,T = T_{\infty } + C_{0} e^{\frac{x}{L}} ,\,\,\,C_{w} = C_{\infty } + C_{0} e^{\frac{x}{L}} .$$

By using Eq. (6), we get:

7 $$\begin{array}{ll} f^{\prime\prime\prime} + ff^{\prime\prime} - 2f^{{\prime}{^{2} }} + 2R_{i} \left( {\theta + N\phi } \right)\cos \alpha + K\left( {6f^{\prime}\,f^{\prime\prime\prime} - 2\eta \left( {f^{\prime\prime}} \right)\,f^{\prime\prime\prime} - 9f^{{\prime\prime}{^{2} }} } \right) \hfill \\ - L\left( {3\left( {f^{\prime\prime}} \right)^{{^{2} }} + \eta \left( {f^{\prime\prime}} \right)\,f^{\prime\prime\prime}} \right) + 3Re\,\beta \left( {f^{\prime\prime}} \right)^{{^{2} }} f^{\prime\prime\prime} - Mf^{\prime} - K^{*} f^{\prime} - Fr\left( {f^{\prime}} \right)^{{^{2} }} = 0, \hfill \\ \end{array}$$

8 $$\left( {1 + Rd} \right)\theta^{\prime\prime} - Prf^{\prime}\theta + Prf\theta^{\prime} + Hs\theta = 0,$$

9 $$\frac{1}{Sc}\phi^{\prime\prime} + f\phi^{\prime} - f^{\prime}\phi - Sc\,R\left( {1 + \delta \Theta } \right)^{n} \varphi \,\,\exp \left( { - \frac{E}{1 + \delta \Theta }} \right) = 0.$$

Transform BCs are:

10 $$\left. \begin{array}{ll} f^{\prime}(\eta ) = 1,\,\,\phi (\eta ) = 1,\,\,\theta (\eta ) = 1,\,\,f(\eta ) = 0\,\,\,\,{\text{as}}\,\,\,\,\eta \to 0 \hfill \\ f^{\prime}(\eta ) \to 0,\,\,\,f^{\prime\prime}(\eta ) \to 1,\,\,\theta (\eta ) \to 1,\,\,\phi (\eta ) \to 0\,\,\,\,{\text{as}}\,\,\,\,\eta \to \infty . \hfill \\ \end{array} \right\}$$

The constraints derived from Eqs. (7)–(9) are given in Table 1.

**Table 1:** The list of dimensionless parameters.

Parameters	Symbols	Expression
Richardson number	*Ri*	$Ri = \frac{Gr}{{{\text{Re}}^{2} }}$
Buoyancy ratio factor	*N*	$N = \frac{{B_{c} C_{0} }}{{{\text{Re}}^{2} }}$
Viscoelastic factor	*L*	$L = \frac{{\alpha_{2} U_{0} e^{\frac{x}{L}} }}{2\rho \nu L}$
Third-grade fluid factor	$\beta$	$\beta = \frac{{\beta_{2} U_{0} e^{\frac{x}{L}} }}{\rho \nu L}$
Cross viscous term	*K*	$K = \frac{{\alpha_{1} U_{0} e^{\frac{x}{L}} }}{{{\text{Re}}^{2} }}$
Permeability factor	K*	$K^{*} = \frac{2\nu L}{{k_{1} U_{0} e^{\frac{x}{L}} }}$
Magnetic factor	*M*	$M = \frac{{2\delta \beta_{0}^{2} }}{{\rho U_{0} }}$
local inertial constant	*Fr*	$Fr = \frac{{2C_{b} L}}{{\sqrt {k_{0} } }}$
Prandtl number	*Pr*	$Pr = \frac{\nu }{\alpha }$
Activation energy	*E*	$E = \frac{{E_{a} }}{{\kappa T_{\infty } }},\,$
Schmidt number	*Sc*	$Sc = \frac{\nu }{{D_{m} }}$
Chemical reaction factor	*R*	$R = \frac{{k_{r}^{2} }}{c}$
Heat source term	*Q*_*e*_	$Q_{e} = \frac{{Q_{0} }}{{\rho C_{p} a}}$
Grashof number	*Gr*	$Gr = \frac{{g\beta_{T} (T_{w} - T_{\infty } )L^{3} }}{2}$
Reynold number	*Re*	$Re = \frac{{U_{0} L}}{\nu }$

The Nusselt number, drag force, Sherwood number are:

11 $$Nu = \frac{{q_{w} x}}{{k\left( {T - T_{\infty } } \right)}},\,\,\,\,C_{f} = \frac{{\tau_{w} }}{{\rho U_{w}^{2} }},\,\,\,\,Sh = \frac{{q_{m} x}}{{D_{m} \left( {C - C_{\infty } } \right)}}.$$

where

12 $$\begin{aligned} \tau_{w} & = \left( {\frac{\partial u}{{\partial y}} + \frac{{\alpha_{1} }}{\mu }\left( {2\frac{\partial u}{{\partial x}}\left( {\frac{\partial u}{{\partial y}}} \right) + u\frac{{\partial^{2} u}}{\partial y\partial x} + v\frac{{\partial^{2} u}}{{\partial y^{2} }}} \right) + \frac{{2\beta_{3} }}{\mu }\left( {\frac{\partial u}{{\partial y}}} \right)^{2} } \right) \\ q_{w} & = - \left( {\frac{\partial T}{{\partial y}}} \right)k,\,\,\,\,q_{m} = - \left( {\frac{\partial C}{{\partial y}}} \right)D_{m} \,\,\,\,\,{\text{at}}\,\,\,\,y = 0. \\ \end{aligned}$$

The dimensionless form of Eq. (11) is:

13 $$\begin{aligned} & C_{fL} = \frac{2}{{\sqrt {Re_{L} } }}\left( {K\left( {3f^{\prime}\left( 0 \right)f^{\prime\prime}\left( 0 \right) - f^{\prime\prime}\left( 0 \right)f\left( 0 \right)} \right) + 2\beta Re_{x} f^{{\prime\prime}{2}} \left( 0 \right) + f^{\prime\prime}\left( 0 \right)} \right), \\ & Re_{L}^{{ - \frac{1}{2}}} Nu_{L} = - \theta \left( 0 \right),\,\,\,Re_{L}^{{ - \frac{1}{2}}} Sh_{L} = - \varphi \left( 0 \right). \\ \end{aligned}$$

## Numerical solution and validation of the problem

The solutions of [Eqs. (7)–(9)] and its BCs [Eq. (10)] are derived in this section. The outcomes are accomplished by engaging the MATLAB code “bvp4c” (built-in package). The bvp4c package is built on the Lobatto III principle^47–49^. Before, solving the Eqs. (7)–(9) and Eq. (10), it must be transformed into first order system of ODEs. The transformation procedure is as follow:

14 $$\Re_{1} = f,\,\,\,\Re_{2} = f^{\prime},\,\,\,\Re_{3} = f^{\prime\prime},\,\,\,\Re_{4} = \theta ,\,\,\,\Re_{5} = \theta^{\prime},\,\,\,\Re_{6} = \phi ,\,\,\,\Re_{7} = \phi^{\prime}.$$

By placing Eq. (14) in Eqs. (7)–(9) and (10) to get:

15 $$\begin{aligned} & \Re^{\prime}_{3} + \Re_{1} \Re_{3} - 2\Re_{2}^{{^{2} }} + 2R_{i} \left( {\Re_{4} + N\Re_{6} } \right)\cos \alpha + K\left( {6\Re_{2} \,\Re^{\prime}_{3} - 2\eta \Re_{3} \,\Re^{\prime}_{3} - 9\Re_{3}^{{^{2} }} } \right) \\ & \quad - L\left( {3\Re_{3}^{{^{2} }} + \eta \Re_{3} \,\Re^{\prime}_{3} } \right) + 3\beta Re\Re_{3}^{{^{2} }} \Re^{\prime}_{3} - K^{*} \Re_{2} - M\Re_{2} - Fr\Re_{2}^{{^{2} }} = 0, \\ \end{aligned}$$

16 $$\left( {1 + Rd} \right)\Re^{\prime}_{5} - Pr\,\Re_{2} \Re_{4} + Pr\,f\Re_{5} + Q_{e} \,\Re_{4} = 0,$$

17 $$\frac{1}{Sc}\Re^{\prime}_{7} + \Re_{1} \Re_{7} - \Re_{2} \,\Re_{6} - Sc\,R\left( {1 + \delta \,\Re_{4} } \right)^{n} \Re_{6} \,\,\exp \left( { - \frac{E}{{1 + \delta \,\Re_{4} }}} \right) = 0.$$

The BCs are:

18 $$\left. \begin{array}{ll} \Re_{1} (\eta ) = 0,\,\,\Re_{2} (\eta ) = 1,\,\,\Re_{4} (\eta ) = 1,\,\,\Re_{6} (\eta ) = 1\,\,\,\,{\text{as}}\,\,\,\,\eta \to 0 \hfill \\ \Re_{2} (\eta ) \to 0,\,\,\,\Re_{3} (\eta ) \to 1,\,\,\Re_{4} (\eta ) \to 1,\,\,\Re_{6} (\eta ) \to 0\,\,\,\,{\text{as}}\,\,\,\,\eta \to \infty . \hfill \\ \end{array} \right\}$$

Table 2 displays the numerical estimation of the present study with the existing works. It has been observed that the present results are precise and consistent.

**Table 2:** Numerical evaluation of the present study with the existing works, while taking $M = 0,\,\,Ri = 0,\,\,L = 0,\,\,Sc = 0,\,\,Fr = 0,\,\,K^{*} = 0.$

*Pr*	Magyari and Keller^50^	Abbas et al.^45^	Present study
1.0	0.9446	0.9452	0.945267
3.0	1.8590	1.8522	1.852283
5.0	2.5100	2.5175	2.517585
10	3.6503	3.6567	3.656832

## Results and discussion

We have calculated the mutual effect of magnetic force and chemical reaction on the energy and mass conduction through the TGF across a stretching sheet.

**Figure 2:**
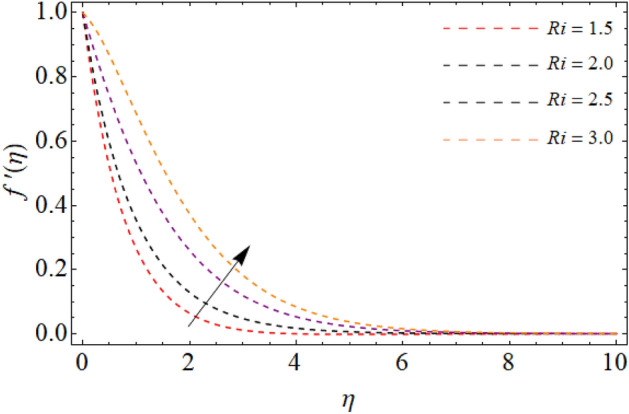
Velocity $f^{\prime}\left( \eta \right)$ versus Richardson number $Ri$.

**Figure 3:**
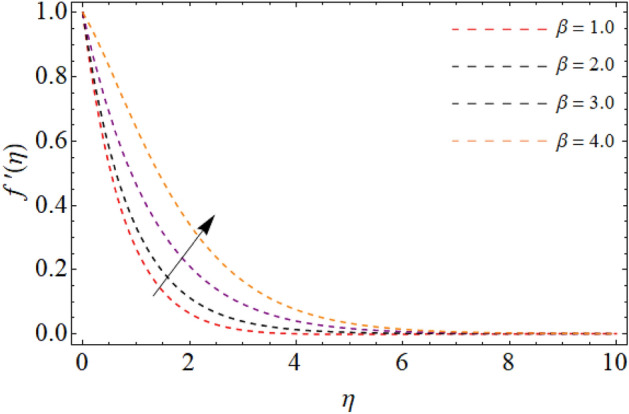
Velocity $f^{\prime}\left( \eta \right)$ versus the third-grade fluid element $\beta .$

**Figure 4:**
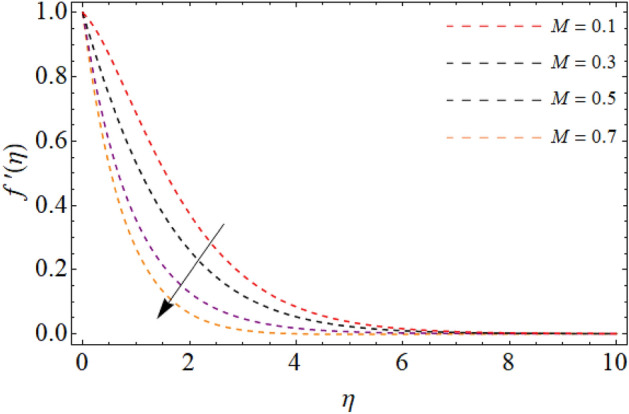
Fluid velocity $f^{\prime}\left( \eta \right)$ versus magnetic term $M$.

**Figure 5:**
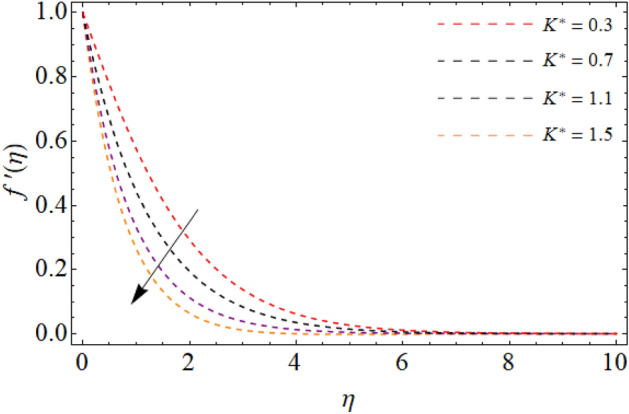
Fluid velocity $f^{\prime}\left( \eta \right)$ versus permeability factor $K^{*}$.

**Figure 6:**
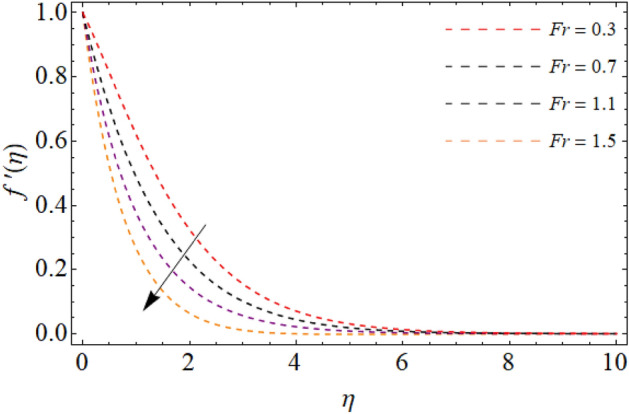
Fluid velocity $f^{\prime}\left( \eta \right)$ versus inertial term *Fr*.

Figures 2, 3, 4, 5 and 6 revealed the effect of Richardson number $Ri$, TGF factor $\beta$, magnetic factor $M$, permeability factor $K^{*}$ and local inertial constant *Fr* versus $f^{\prime}\left( \eta \right)$. Figures 2 and 3 reports that the velocity curves develop for the rising values of Richardson number $Ri$ and third-grade fluid factor. Richardson number is the ration between Grashof and Reynold number. The Reynold number has an transposed relation with *Ri*, therefore the fluid velocity $f^{\prime}\left( \eta \right)$ improves with the variation of *Ri*. Similarly, the action of third-grade fluid factor $\beta$ also enhances the velocity as presented in Fig. 3. Physically, the kinetic viscosity drops, while the stretching velocity of fluid develops with the effect of $\beta$, which results in such scenario. The influence of magnetic factor $M$, permeability factor $K^{*}$ and local inertial constant *Fr,* all diminish the fluid velocity as publicized in Figs. 4, 5 and 6. Physically, the resistive force opposes the fluid velocity $f^{\prime}\left( \eta \right)$, which is produced due to magnetic effect (Fig. 4). On the other hand, the rising permeability of the sheet resists to the flow field, which causes in the reducing of velocity field (Fig. 5). The consequences of inertial forces also decline the velocity curve $f^{\prime}\left( \eta \right)$ as exposed in Fig. 6.

**Figure 7:**
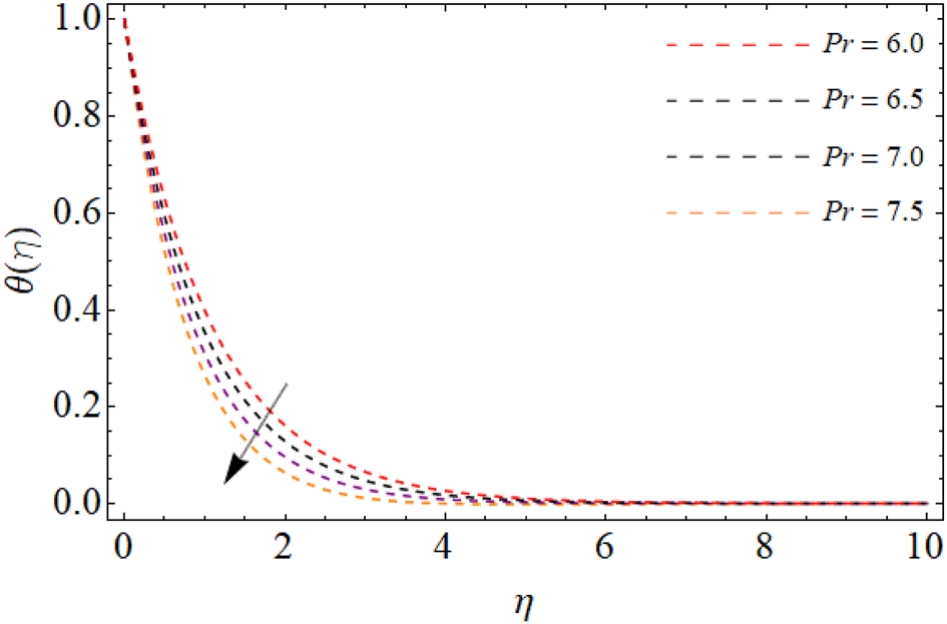
Fluid energy sketch $\theta \left( \eta \right)$ versus Prandtl number *Pr*.

**Figure 8:**
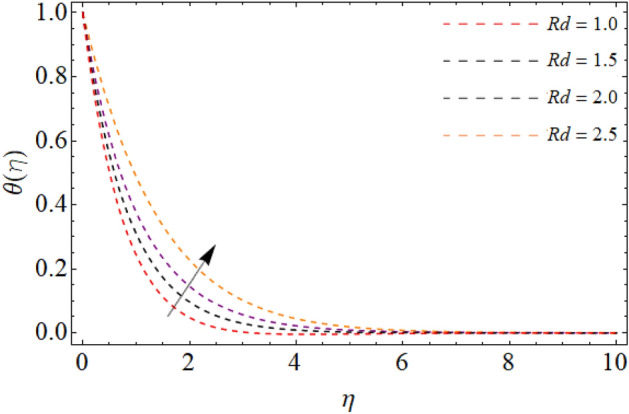
Fluid energy sketch $\theta \left( \eta \right)$ versus thermal radiation *Rd*.

**Figure 9:**
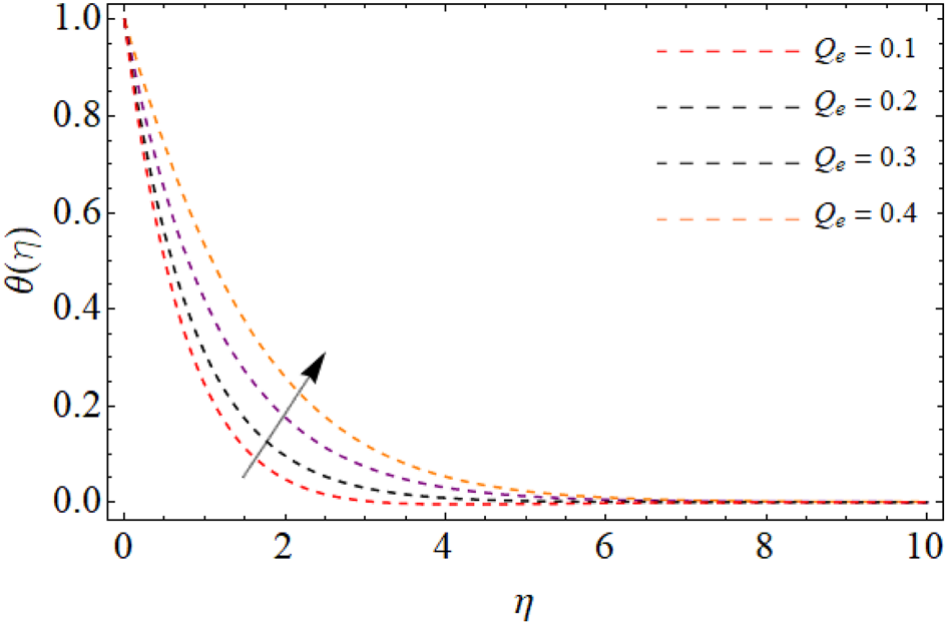
Fluid energy sketch $\theta \left( \eta \right)$ versus Heat source $Q_{e}$.

**Figure 10:**
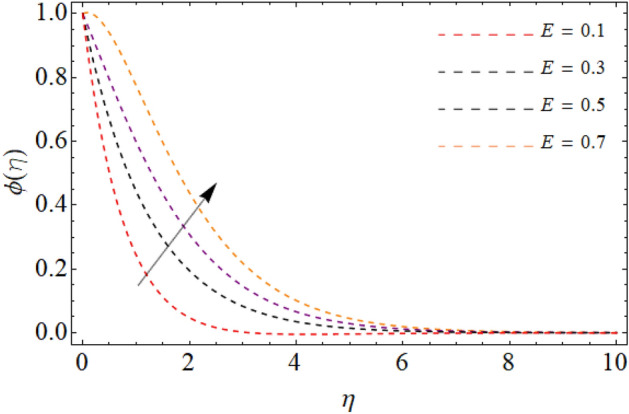
Fluid concentration sketch $\phi \left( \eta \right)$ versus Activation energy *E*.

**Figure 11:**
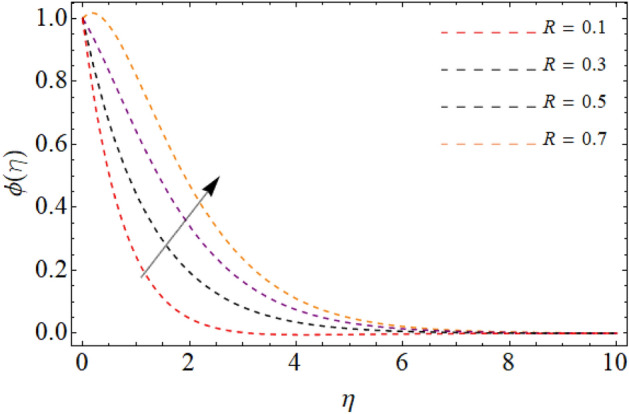
Fluid concentration sketch $\phi \left( \eta \right)$ versus chemical reaction *R*.

**Figure 12:**
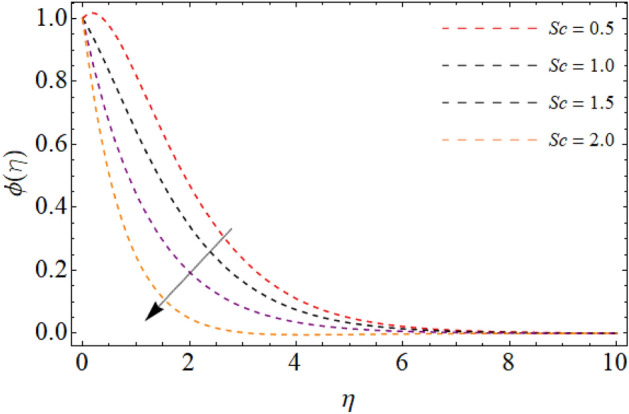
Fluid concentration sketch $\phi \left( \eta \right)$ versus Schmidth number *Sc*.

Figures 7, 8 and 9 highlight the significances of Prandtl number, *Rd* and $Q_{e}$ on the energy $\theta \left( \eta \right)$ field. Figure 7 exposes that the temperature curve drops with the effect of Prandtl number. Physically, the thermal diffusivity of higher Prandtl fluid is less, that’s why, the effect of *Pr* drops the energy field (Fig. 7). The radiation effect transfers thermal energy form heat source to the system, which results in the elevation of temperature field $\theta \left( \eta \right)$ (Fig. 8). Similarly, the heat source working as heating agent for the flow system, there effect rises the fluid temperature $\theta \left( \eta \right),$ as displayed in Fig. 9. Figures 10 and 11 highlights the significances of activation energy *E*, *R* and Schmidth number *Sc* on the mass profile $\phi \left( \eta \right)$. Figures 10 and 11 explained that activation energy factor and chemical reaction effect augments the concentration field. Correspondingly, the significance of *Sc* controls the mass transfer, because the kinetic viscosity improves, which lessens the mass $\phi \left( \eta \right)$ outline as discovered in Fig. 12. The amount of the chemical reaction has a direct impact on the intensity of mass transfer, because it makes fluid atoms move more quickly, which causes the mass gradient $\phi \left( \eta \right)$ to rise as publicized in Fig. 11.

Table 3 disclosed the numerical outputs for skin friction $f^{\prime\prime}\left( 0 \right)$, Sherwood number $- \phi^{\prime}\left( 0 \right)$ and Nusselt number $- \theta^{\prime}\left( 0 \right)$. It has been noticed that the Nusselt number and Skin friction rises for the mounting values of Schmidth number.

**Table 3:** The numerical outputs for skin friction $f^{\prime\prime}\left( 0 \right)$, Sherwood number $- \phi^{\prime}\left( 0 \right)$ and Nusselt number $- \theta^{\prime}\left( 0 \right)$.

*N*	$f^{\prime\prime}\left( 0 \right)$	$- \theta^{\prime}\left( 0 \right)$	$- \phi^{\prime}\left( 0 \right)$
0.3	1.5047730457472	2.7930245333898	0.3804378693486
0.5	0.6521245649586	3.0398628093226	0.4354683514387
0.7	− 0.5760489752623	3.3020406132527	0.4897833078565
0.9	− 1.3605022868920	3.4249212406151	0.5160047901888
*Sc*	–	–	–
0.1	1.5047730547472	2.7930245333898	0.3804378693486
0.2	1.5153784960713	2.7861051786865	1.3656576445162
0.3	1.5244263887091	2.7814596692455	2.3837736504415
0.4	1.5280410380701	2.7799706702532	2.8994715445831

## Conclusions

We have numerically calculated the energy and mass transmission through the third‐grade fluid and relation of the Darcy–Forchheimer across a stretching sheet. Additionally, the consequences of heat source, thermal radiation and magnetic effect are also studied with the fluid flow. The simplified set of ODEs is numerically resolved through the bvp4c technique, by using Mathematica software. The main findings are:

- Velocity curve enhances for the rising values of Richardson number $Ri$ and third-grade fluid factor.
- The influence of magnetic factor $M$, permeability factor $K^{*}$ and local inertial constant *Fr,* all diminish the fluid velocity.
- The temperature curve drops with the effect of Prandtl number.
- The radiation effect transfers thermal energy form heat source to the system, which results in the elevation of energy field $\theta \left( \eta \right)$.
- The heat source working as heating agent for the flow system, there effect rises the fluid temperature $\theta \left( \eta \right)$.
- The consequence of chemical reaction boosts the concentration field, while declines with the Schmidth number.

## Data Availability

All data used in this manuscript have been presented within the article.

## List of symbols

$U_{w}$: Sheet stretching velocity
$$qr$$: Thermal radiation
$$K_{0}$$: Surface penetrability
*Ea*: Activation energy
*F*: Inertia constant
$$\nu$$: Kinematic viscosity
$$\alpha_{m}$$: Thermal diffusivity
$$\mu$$: Dynamic viscosity
$$D_{m}$$: Mass diffusivity
*N*: Buoyancy ratio factor
*M*: Magnetic factor
*g*: Gravitational acceleration
*T*_*w*_: Surface temperature
NN: Non-Newtonian
$$\delta$$: Electrical conductivity
($$\alpha_{1}$$,$$\beta_{3}$$,$$\alpha_{2}$$): Material moduli
$$Q_{0}$$: Heat source
$$B(x)$$: Magnetic field
$C_{p}$: Specific heat competence
$$k_{r}$$: Chemical reaction
*Ri*: Richardson number
*L*: Viscoelastic factor
*K*^***^: Permeability factor
$\beta$: Third-grade fluid factor
*R*: Chemical reaction factor
*C*_*w*_: Surface concentration
MHD: Magnetohydrodynamics
TGF: Third grade fluid
